# Fifteen Thousand Pounds for the Charities

**Published:** 1895-11-02

**Authors:** 


### Fifteen Thousand Pounds for the Charities.

The Lord Mayor has distributed the £15,000 given by Mr. Daniel Marks, of Messrs. Marks, Bulteel, Mills, and Co., on behalf of Messrs. B. I. Barnato and several other City friends, for distribution among the charitable institutions of the metropolis, as follows: (1) To the Hospital Sunday Fund, in connection with the special appeal made by The Hospital, £10,000; (2) the poor-boxes of the City and metropolitan police-courts, to be divided according to the Lord Mayor's discretion, £2,000; (3) the Mansion House Committee on the Problem of the Poor in London, to enable them to train friendly workers, £2,000; (4) the Hospitals Association, in trust to establish a reference library on all subjects connected with hospitals and charities generally, £600; and (5) the Charity Organisation Society, pension cases and other objects, as per list enclosed, £400.

